# Eye-Inspired Single-Pixel Imaging with Lateral Inhibition and Variable Resolution for Special Unmanned Vehicle Applications in Tunnel Inspection

**DOI:** 10.3390/biomimetics9120768

**Published:** 2024-12-18

**Authors:** Bin Han, Quanchao Zhao, Moudan Shi, Kexin Wang, Yunan Shen, Jie Cao, Qun Hao

**Affiliations:** 1 School of Optics and Photonics, Beijing Institute of Technology, Beijing 100081, China; 3120170330@bit.edu.cn (B.H.); 3220220495@bit.edu.cn (M.S.); 3120240572@bit.edu.cn (Y.S.); qhao@bit.edu.cn (Q.H.); 2 Yangtze Delta Region Academy of Beijing Institute of Technology, Jiaxing 314019, China; zqc@bitjx.edu.cn; 3 The China Railway 12th Bureau Group Company, Ltd., Taiyuan 030027, China; tiehang@yftong.com.cn; 4 Changchun University of Science and Technology, Changchun 130022, China

**Keywords:** single-pixel imaging, variable resolution, lateral inhibition, tunnel inspection, unmanned vehicles

## Abstract

This study presents a cutting-edge imaging technique for special unmanned vehicles (UAVs) designed to enhance tunnel inspection capabilities. This technique integrates ghost imaging inspired by the human visual system with lateral inhibition and variable resolution to improve environmental perception in challenging conditions, such as poor lighting and dust. By emulating the high-resolution foveal vision of the human eye, this method significantly enhances the efficiency and quality of image reconstruction for fine targets within the region of interest (ROI). This method utilizes non-uniform speckle patterns coupled with lateral inhibition to augment optical nonlinearity, leading to superior image quality and contrast. Lateral inhibition effectively suppresses background noise, thereby improving the imaging efficiency and substantially increasing the signal-to-noise ratio (SNR) in noisy environments. Extensive indoor experiments and field tests in actual tunnel settings validated the performance of this method. Variable-resolution sampling reduced the number of samples required by 50%, enhancing the reconstruction efficiency without compromising image quality. Field tests demonstrated the system’s ability to successfully image fine targets, such as cables, under dim and dusty conditions, achieving SNRs from 13.5 dB at 10% sampling to 27.7 dB at full sampling. The results underscore the potential of this technique for enhancing environmental perception in special unmanned vehicles, especially in GPS-denied environments with poor lighting and dust.

## 1. Introduction

In the realm of public safety and infrastructure maintenance, the deployment of special unmanned vehicles (UAVs) has become increasingly vital, particularly in GPS-denied environments such as tunnel inspection and bridge monitoring [1,2,3]. These operations, which are essential for public safety, require frequent and accurate assessments to ensure the integrity and safety of critical infrastructure. However, traditional imaging techniques often struggle in complex environments characterized by poor lighting and the presence of dust [1,4]. The constraints on the size and weight of imaging systems for unmanned vehicles further exacerbate these challenges, demanding a novel approach to optical imaging that is both compact and powerful [5].

Single-pixel imaging (SPI) has emerged as an innovative solution to these limitations, offering structural simplicity, resolution independence, and high sensitivity [6,7,8]. This technology is particularly adept at operating in low-light conditions, presenting a promising new path for enhancing the environmental perception capabilities of UAVs. To address the challenges faced in tunnel inspection, we have partnered with the China Railway 12th Bureau Group Company, Ltd., to develop an eye-inspired single-pixel imaging system with lateral inhibition and variable resolution. This system is designed to significantly enhance a vehicle’s environmental perception capabilities in challenging environments.

Our approach integrates variable-resolution imaging with lateral inhibition, aiming to improve the efficiency and quality of the image reconstruction of fine targets in practical applications. This technique mimics the human eye’s ability to concentrate high-resolution vision only in the region of interest, offering significant advancement in the field of imaging for unmanned vehicle navigation and inspection tasks. By emulating the high-resolution foveal vision of the human eye, this method significantly enhances the efficiency and quality of image reconstruction for fine targets within the region of interest (ROI). This method utilizes non-uniform speckle patterns coupled with lateral inhibition to augment optical nonlinearity, leading to superior image quality and contrast. Lateral inhibition effectively suppresses background noise, thereby improving the imaging efficiency and substantially increasing the signal-to-noise ratio (SNR) in noisy environments [2,9].

The concept of single-pixel imaging is not new; it has been explored extensively in the field of quantum imaging and has recently transitioned to classical computational imaging [6,10,11]. The unique advantage of GI lies in its ability to separate the detection of light from the formation of an image, allowing the use of a single-pixel detector and enabling high-speed, high-resolution imaging [12,13,14]. This is particularly beneficial in scenarios where the imaging environment is hostile or where the target is moving rapidly [7,8,15].

In this study, we present a cutting-edge imaging technique that integrates single-pixel imaging inspired by the human visual system with lateral inhibition and variable resolution to improve environmental perception in challenging conditions, such as those with poor lighting and dust. By emulating the high-resolution foveal vision of the human eye, this method significantly enhances the efficiency and quality of image reconstruction for fine targets within the region of interest (ROI). This method utilizes non-uniform speckle patterns coupled with lateral inhibition to augment optical nonlinearity, leading to superior image quality and contrast. Lateral inhibition effectively suppresses background noise, thereby improving the imaging efficiency and substantially increasing the signal-to-noise ratio (SNR) in noisy environments [2,9].

The structure of this paper is as follows: The subsequent section delves into the principle of our eye-inspired GI, detailing the theoretical underpinnings and the innovative aspects of our approach. We then describe the methods employed in our study, including the experimental setup and the protocol for data acquisition and analysis. The results section presents the findings from our indoor experiments and field tests, demonstrating the performance of our method under controlled conditions and in real-world tunnel scenarios. Finally, we conclude with a discussion of the implications of our results, the potential applications of this technology, and the future directions of our research.

Through this work, we aim to contribute a reliable imaging solution that enhances safety and efficiency in tunnel inspections and other applicable domains, marking a significant advancement over traditional imaging techniques. Our research underscores the potential of bio-inspired computational imaging to overcome the limitations of current technologies and to meet the demanding requirements of special unmanned vehicles in GPS-denied environments.

## 2. Methods

### 2.1. Principle

**Figure 1:**
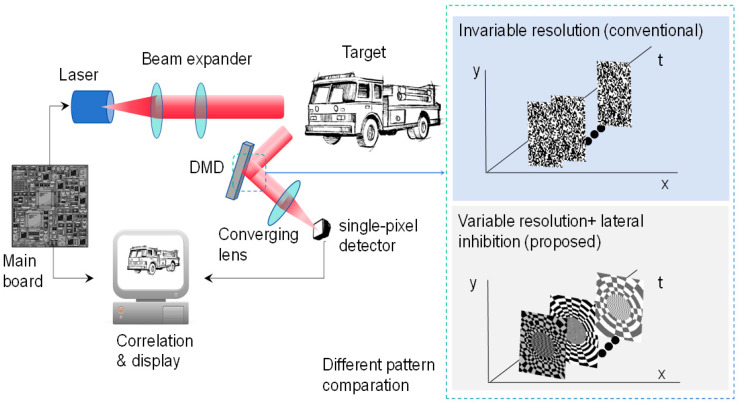
The principle of eye-inspired GI.

The principle of eye-inspired GI [16,17] is shown in Figure 1, in which the laser is triggered by the main board and illuminates the target through a beam expander. The light is then reflected from the target and modulated by the digital mirror device (DMD) [18,19]. The DMD is loaded with our pre-designed variable-resolution speckles. The square where the target object is located is modulated by a variable-resolution speckle. A single-pixel detector receives light for modulation [20,21]. Finally, the target image is reconstructed and displayed by performing a cross-correlation operation [22] between the light intensity signals and the light field information modulated by the DMD.

From the principle above, the marketable difference of our method is the use of non-uniform patterns combined with the feature of lateral inhibition. Therefore, the advantages of the proposed method include the following: (1) Enhanced Image Quality: The non-uniform speckle pattern increases the optical nonlinearity of the imaging system, which aids in capturing more details and contrast during the imaging process, thereby enhancing image quality. (2) Improved Imaging Efficiency: The lateral inhibition mechanism suppresses background noise and enhances the useful signal, thus improving the imaging efficiency of the system. (3) Improved noise resistance: The lateral inhibition mechanism helps reduce noise interference during the imaging process, particularly in complex or noisy imaging environments; thus, it can significantly enhance the SNR of the imaging.

### 2.2. Variable-Resolution Projection Pattern

In this study, a new variable-resolution projection pattern is used to replace the traditional fixed-resolution projection pattern. The design of this new pattern is inspired by the non-uniform distribution pattern of receptors on the retina of the human eye, where the projection area is divided into a high-resolution fovea region and a low-resolution marginal region. In the fovea region, we use the standard uniform high-resolution Cartesian sampling method, which avoids the oversampling problem that can occur when using log-polar coordinates in the central region. In the edge region, we use the logarithmic polar coordinate variable-resolution sampling technology, using the characteristics of this coordinate system to compress the image properly. With this design, we successfully simulated the imaging characteristics of the human eye, which provides a high-resolution image in the central area of the field of view and a low-resolution image in the peripheral area of the field of view.

**Figure 2:**
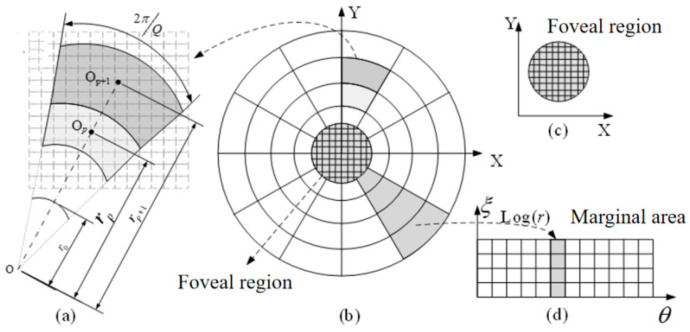
Variable-resolution projection pattern structure diagram.

The variable-resolution projection pattern consists of two parts, the foveal region and the edge region, as shown in Figure 2. Figure 2a shows a detailed enlarged view of the edge region; Figure 2b shows the entire variable-resolution projection pattern, including the foveal region and the marginal region; Figure 2c specifically shows the foveal area; Figure 2d shows the marginal region. In the fovea, high-resolution sampling is generated using the traditional Cartesian coordinate system, which avoids the oversampling problem that occurs when using log-polar coordinates in the fovea. The edges are based on a log-polar model that mimics the human eye. If r is used to represent the distance from the pixel point to the center point, then the outer boundary of the foveal region, that is, the ring with radius r_0_, belongs to the foveal region; and the part outside this ring is the edge region.

According to the difference between the polar diameter and the polar angle, the edge region can be divided into *P* rings; each ring has *Q* pixels. In the human-simulated log-polar model, let *p* and *q* represent the *p*-ring and QTH pixel, respectively; then, the variable-resolution structure of the edge region can be calculated by the following equations:

(1) rp+1=r1 · ℇpℇ=1+sin⁡πQ1−sin⁡πQr1=r01−sin⁡πQθq=q ·2πQ           q=1,2,3…Qξ=logεrp=logεr1+p−1           (p=1,2,3…P)

where *r_p_* represents the radius of the ring where the PTH ring pixel is located, *θ_q_* represents the angle corresponding to the *q* pixel, and *ε* represents the growth coefficient between the rings.

The steps to achieve the variable-resolution projection pattern are as follows: First, set the radius of the foveal area *r*_0_ as a reference. Then, according to the distance between each pixel and the center point, the whole projection pattern is divided into the foveal region and the variable-resolution edge region. In the foveal region, we divide it evenly into *P_f_* pixels according to the conventional method. For the variable-resolution edge region, we calculate the additional parameters required for generation according to specific constraints. For example, if the constraint is the number of pixels per ring *Q*, then we calculate the growth coefficient between rings *ε*, the radius of each ring rp, and the number of rings *P* based on this condition. Then, Formula (1) is used to generate *P_p_* pixels of the edge region. Similarly, other required parameters can be calculated from the number of rings *P*. Finally, a complete variable-resolution projection pattern is obtained by merging the foveal region with the variable-resolution edge region.

### 2.3. The SPI with Variable Resolution and Lateral Inhibition

The core theoretical contribution of this method is the integration of variable resolution and lateral inhibition into a single-pixel imaging model. The model mimics the foveated vision of the human eye, where high-resolution imaging is reserved for the region of interest, whereas the periphery is imaged at a lower resolution [23,24]. Meanwhile, lateral inhibition is incorporated to enhance contrast and detail in the final image reconstruction. The image reconstruction based on the SPI pattern model of variable resolution and lateral inhibition includes six steps.

Step 1: Variable resolution modulation function

Variable Resolution Concept: Variable resolution adjusts imaging detail based on the importance of different areas within the field of view (FOV), with high resolution for the region of interest (ROI) and lower resolution for the peripheral areas. The modulation function *M*(*x*, *y*) is expressed as:

(2) $$M\left(x,y\right)=exp\left[-\frac{d^{2}(x,y)}{2\sigma^{2}}\right]$$

where *d*(*x*, *y*) is the distance from point (*x*, *y*) to the center of the FOV, and *σ* is a decay parameter that controls the sharpness of the transition between high- and low-resolution areas.

Step 2: Modulation of the Speckle Pattern

Based on Step 1, the integration of the variable resolution *P*(*x*, *y*) is written as

(3) $$P\left(x,y\right)=P_{0}(x,y)\cdot M(x,y)$$

where *P*_0_(*x*, *y*) is the initial uniform speckle pattern and *M*(*x*, *y*) modulates this pattern based on the variable resolution requirement, thereby enhancing the resolution of the ROI.

Step 3: Application of Lateral Inhibition

We introduce the lateral inhibition function *S*(*x*, *y*), which is written as

(4) Sx,y=      if x,y is in the ROIotherwiseε1

where *ε* is a small positive number. This function is crucial for enhancing the contrast by reducing the effect of the peripheral region.

Step 4: Generation of the Detected Beam

The intensity of the detected beam’s intensity *D*(*x*, *y*) after passing through the target is given by

(5) $$D\left(x,y\right)=\iint P(x^{\prime },y^{\prime })\;\cdot I\left(x^{\prime }-x,\;y^{\prime }-y\right)dx^{\prime }dy^{\prime }$$

where *P*(*x*′, *y*′) is the modulated speckle pattern that considers both variable resolution and lateral inhibition, and *I*(*x*′ − *x*, *y*′ − *y*) is the intensity distribution of the target.

Step 5: Calculation of the second-order correlation function

The second-order correlation function *G*^2^(*τ*) is used in the imaging process and is written as

(6) $$G^{2}\left(\tau \right)=\langle I_{ref}\left(t\right)I(t+\tau )\rangle$$

This function measures the correlation between the reference beam and the detected beam, which is influenced by the modulated speckle pattern.

Step 6: Image Reconstruction

(7) $$I^{\prime }(x,y)\propto \iint G^{2}\left(\tau \right)\cdot [P_{0}(x^{\prime }+x,\;y^{\prime }+y)\cdot M(x^{\prime },y^{\prime })\cdot S(x^{\prime },y^{\prime })]\times dx^{\prime }dy^{\prime }$$

The reconstructed image *I*′(*x*, *y*) is proportional to the integral of the product of the second-order correlation function and the complex conjugate of the modulated speckle pattern, which includes both variable-resolution and lateral inhibition effects.

From the above processes, by integrating the variable-resolution model more explicitly into the derivation, we enhance the clarity of how different areas within the FOV are treated based on their importance. This approach ensures that the ROI is imaged with a higher resolution and contrast, while peripheral areas are adjusted to reduce noise and interference, leading to a more effective single-pixel imaging technique.

According to the size of the region of interest and the region of non-interest, the log-polar model can be used to generate the spatial variable-resolution sampling structure with different position resolutions. By using the side suppression feature, the projection pattern can be optimized based on the boundary and detail characteristics of the obtained imaging results. In traditional ghost imaging, random speckles or stripes based on image transformation basis are used as projection patterns. However, the characteristics of the current imaging scene are not considered, especially for the low-contrast scene which is poorly imaged by the existing methods. The method proposed in this paper establishes the connection between the scene information and the projection pattern by combining the side suppression feature with the variable-resolution speckle and adaptively adjusts the projection pattern according to the characteristics of the current scene, which can effectively enhance the edge details and improve the contrast and image quality.

## 3. Experiments and Results

The experiments were designed to validate two critical aspects of the proposed method: the compression of redundant data and the ability to capture clear images under actual working conditions. To address these objectives, both indoor and field experiments were conducted. The indoor experiments utilized discrete optical components to validate the advantages of the compression of redundant data, whereas the field experiments were conducted in real tunnel scenarios to mimic real-world operational challenges.

### 3.1. Experimental Setup

**Figure 3:**
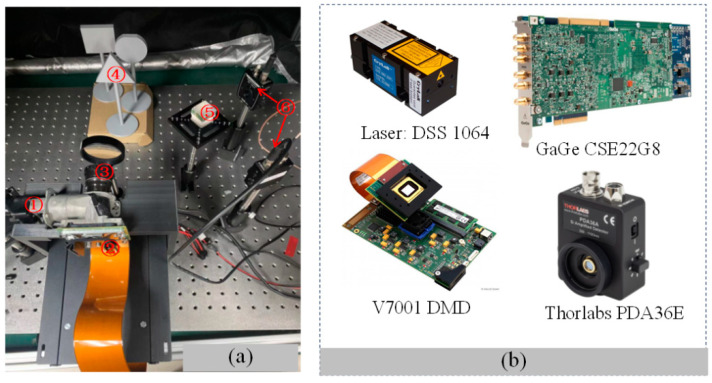
Indoor experimental setup. (**a**) The structure of the experiment; (**b**) the main components used in the indoor experiments.

In the indoor experiments, as shown in Figure 3, we assembled a high-performance system that corresponded to the components diagrammatically represented in the provided image. The heart of our setup was a pulse laser (①) from the DSS 1064 series of the CryLas laser system, selected for its stability and precision to ensure reliable light emission. This was complemented by a Vialux V7001 DMD (②), chosen for its 1024 × 768 resolution and 22 kHz micromirror flip rate, to generate and modulate speckle patterns. The DMD was securely mounted adjacent to the pulse laser, with light-shielding tape applied to prevent stray light interference. Our transmission optical component (③) consisted of a series of precision optical elements, such as lenses and beam shapers that direct and focus the speckle pattern onto the detection target (④) [25,26]. The targets, including triangular, circular, and square shapes, were fabricated using 3D printing technology to construct a 3D scene for imaging, simulating potential tunnel-inspection scenarios. The light reflected from the targets was directed to two single-pixel photodiodes (⑥) [16,27] via a beam splitter (⑤). The photodiode (Thorlabs’ PDA36E, Sorebo Optoelectronic Technology Shanghai, China), recognized for its high sensitivity and wide bandwidth, effectively captured the nuances of the light signals. All these components were mounted on custom 3D-printed bases to ensure accurate alignment. To manage the overall timing of the system, which is crucial for measuring the time intervals of the reflected light signals and facilitating the reconstruction of single-pixel images, we employed a field-programmable gate array (FPGA)-based time-to-digital converter (TDC) system. A data acquisition card (GaGe CSE22G8) was used to collect and digitize the analog signals from the photodiodes, which were then transferred to a computer for further processing and analysis. This comprehensive setup, with its carefully selected and integrated components, allowed us to effectively evaluate the variable-resolution imaging technique under controlled conditions, demonstrating its potential to enhance image quality, especially in capturing fine details within the ROI.

In the indoor experiments, we utilized a setup that included geometric targets, such as a triangular prism with a 10 cm side length, a circular disc with a 10 cm diameter, and a square prism with a 10 cm side length. These targets were strategically positioned 60 cm from the light source.

In the field experiments, we aimed to address the practical challenges of tunnel inspection by collaborating with the China Railway Twelve Bureau to develop a specialized integrated land and air inspection robot. The robot was designed to navigate and inspect the interior walls of tunnels according to pre-planned paths and to transmit the collected inspection data to a terminal for analysis by professionals. Given that the tunnels under inspection are not in their final state, the robot operates in harsh environments characterized by GPS signal blockage, dim lighting, and complex obstacles on the tunnel walls. These conditions make it difficult for the robot to avoid obstacles; the typical obstacles include wires, cables, and angle irons. In the experiments, we selected a cable of 0.5 cm as the target. While existing 3D imaging LiDAR can map the interior of a tunnel and roughly locate larger obstacles, it struggles to accurately image small, fine details; such imaging is crucial for precise localization. Additionally, the size, weight, and power consumption of current LiDAR systems make it challenging to integrate them directly into special unmanned vehicles. We focused on field experiments that imaged fine targets under actual working conditions to guide and enhance the environmental perception capabilities of special unmanned vehicles.

**Figure 4:**
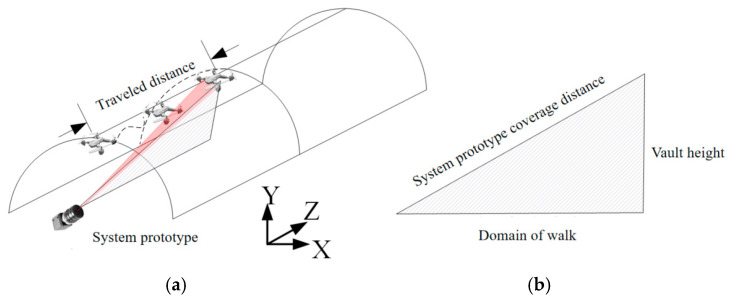
Schematic diagram of the working distance of the inner wall of the robot tunnel. (**a**) Working distance diagram; (**b**) range of coverage.

Taking into account the actual working environment, the typical walking distance of the UAVs from the beginning to the detection is 100 m, as shown in Figure 4. The arch height of the railway tunnel in China is different according to the actual situation; for example, the height from the rail surface to the arch of the railway tunnel of the Guiguan Line is 8.68 m. Meanwhile, according to the Design Code for High-Speed Railway Tunnels, it is required to be no less than 8 m, and 10 m is taken for the calculation. Therefore, the covering distance of the system prototype should be no less than 100.5 m. In order to complete the laboratory experiment, the system was integrated, as shown in Figure 3a, and the system test environment is shown in Figure 3b.

**Figure 5:**
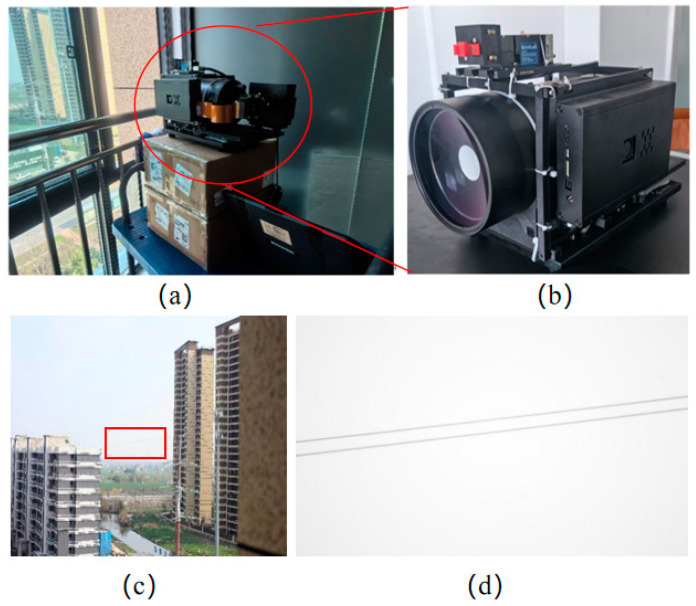
Setup of the infield experiment. (**a**) The location of the laboratory equipment; (**b**) enlarged view of the experimental setup; (**c**) the environment of the target object; (**d**) target object.

We carried out an infield experiment, in which the experimental equipment was placed inside the house; the specific location and system prototype are shown in Figure 5a,b. Two cables were placed 100 m away from the system; the test environment is shown in Figure 5c. The diameter of the cable was 0.5 cm, as shown in Figure 5d.

**Figure 6:**
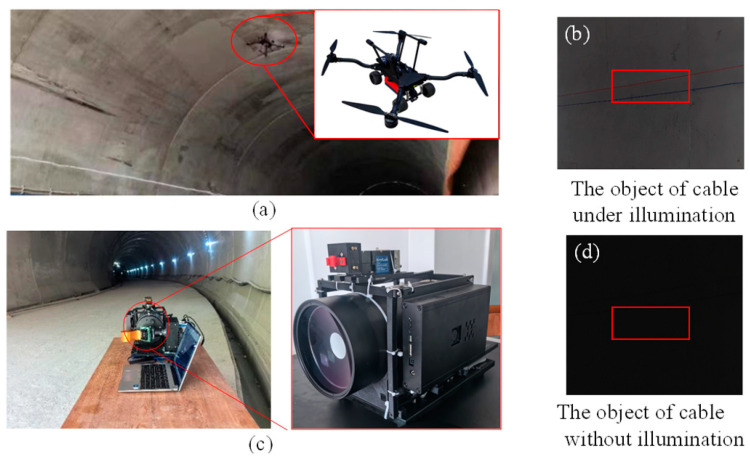
Field experimental setup. (**a**) Specialized robot under working conditions. (**b**,**d**) are the target of cable under illumination and without illumination. (**c**) The prototype is displaced at the tunnel entrance.

The prototype, as depicted in Figure 6c, integrates a 120 mm aperture catadioptric telescope for long-range single-pixel imaging of 100 m, enhancing the ability to detect small targets at a distance. Given the harsh conditions in a tunnel, such as dim lighting and the presence of dust, traditional imaging methods struggle to produce clear images. As shown in Figure 6b, even with a mobile phone flash at approximately 5 m, the visibility is significantly limited, and without illumination, as seen in Figure 6d, the cables are nearly indistinguishable to the naked eye. The core components of the rest of the system, including the pulse laser, DMD, and single-pixel detectors, are consistent with those used in our indoor experiments, ensuring a reliable baseline for comparison.

### 3.2. Results and Discussion

Our experiments aimed to validate the performance of the proposed eye-inspired variable-resolution single-pixel imaging technique under controlled indoor conditions and real-world field scenarios.

#### 3.2.1. Data Compression Performance Verification (Indoor Experiments)

**Figure 7:**
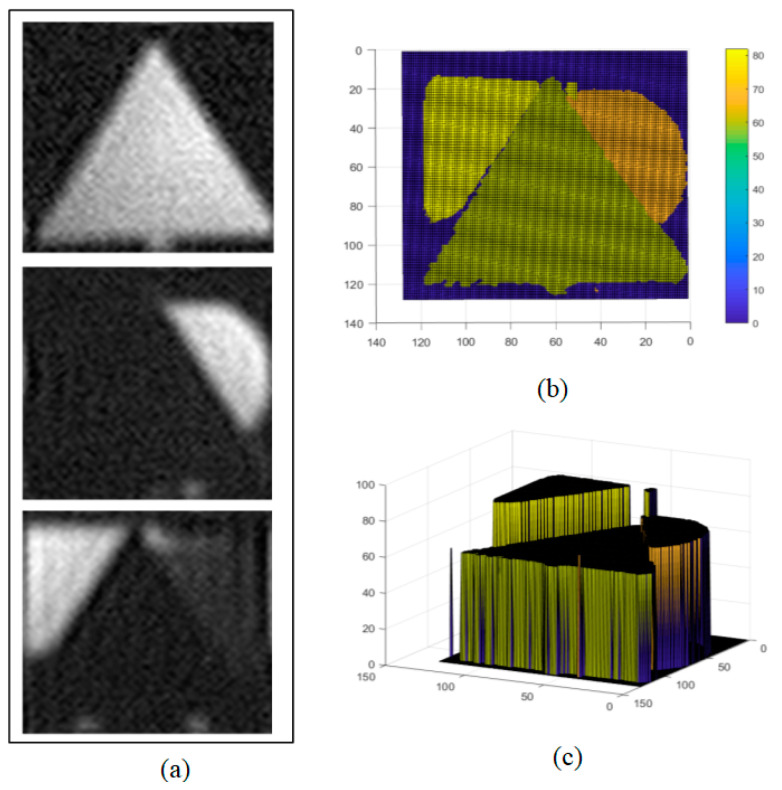
Traditional uniform-resolution random speckle 3D imaging results. (**a**) Reconstructed intensity map, (**b**) reconstructed distance map, and (**c**) 3D view.

To compare the imaging effects, we used three basic geometric shapes: an equilateral triangle, a circle, and a square, each with a side length or diameter of 10 cm, placed at intervals of 8 cm and 14 cm from each other. The triangle was positioned 60 cm from the light source, covering the entire FOV, which was defined as the region of interest (ROI). We captured data with 2500 samples; the imaging results are shown in Figure 7, where (a) represents the reconstructed intensity maps at different distances, (b) shows the distance maps in different colors, and (c) presents a three-dimensional view.

**Figure 8:**
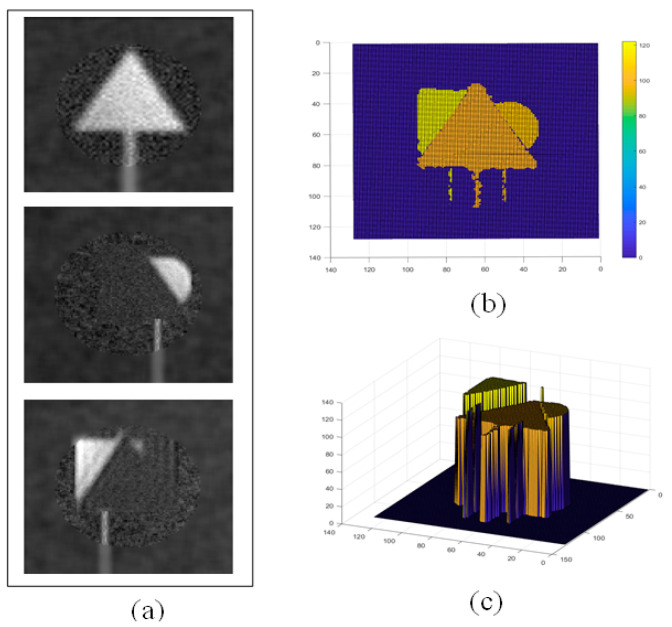
Three-dimensional imaging results using eye-inspired variable-resolution speckle method. (**a**) Reconstructed intensity map, (**b**) reconstructed distance map, and (**c**) 3D view.

Compared to the traditional uniform random speckle pattern reconstruction methods, our experiment applied a variable-resolution sampling technique, reducing the number of samples by half. This improvement demonstrates an increase in the reconstruction efficiency while maintaining the image quality, highlighting the potential of optimizing the reconstruction process in GI technology by adjusting the sampling strategy. When the distance between the triangular target and light source was increased to 100 cm, occupying only a part of the imaging FOV, we optimized the imaging effect by selecting the central area of the FOV as the ROI. By mimicking the retinal structure of the human eye, we generated a high-resolution speckle pattern in this area. The central area, with a radius of 42 pixels, was surrounded by five concentric circular rings, each containing 72 pixels, totaling 4432 pixels. The central region was filled with variable-resolution speckles, similar to the human eye, while the peripheral area was filled with random speckles. Even when the sample number was reduced to 1500, which was only 0.1 of the traditional uniform speckle sampling rates, the reconstructed image still achieved good quality, as shown in Figure 8. This result confirms that with an optimized speckle pattern, clear imaging results can still be obtained even at a reduced sampling ratio, thereby effectively verifying the characteristic of variable resolution in compressing redundant data.

#### 3.2.2. Fine Target Imaging Capability Verification

**Figure 9:**
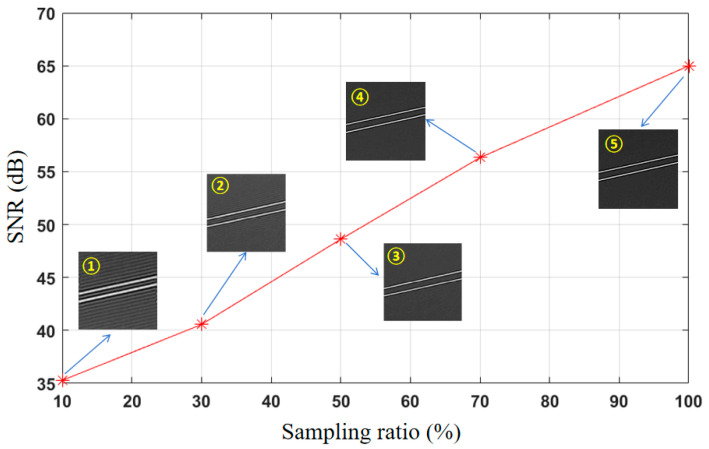
SNRs under different sampling ratios. The sampling ratios from ① to ⑤ were 10, 30, 50, 70, and 100%, respectively.

Two cables were placed 100 m away from the system to image them, and the reconstruction quality was compared under different association times, as shown in Figure 9, which shows the system imaging SNR with the system sampling rate ranging from 10% to 100%. It can be seen from the figure that even if the sampling rate is reduced to 10%, the resulting image is still able to maintain a signal-to-noise ratio of up to 35 dB. This shows that even under the condition of a low sampling rate, the system constructed in this paper can still obtain high-quality images, so as to support the UAVs and help them to more accurately perceive the environment and avoid obstacles.

**Figure 10:**
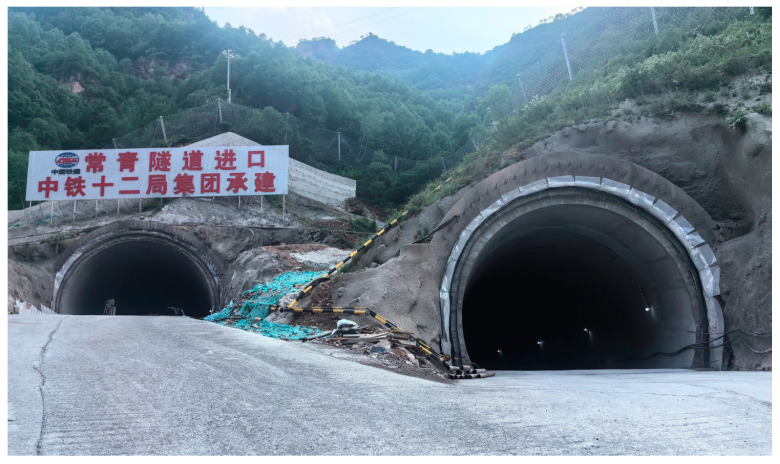
A section of closed tunnel in Xiongqin (Xiongan to Qinzhou).

After verifying the feasibility of the system prototype at the simulated site, the system prototype was deployed to the actual application environment, and the site was selected as a section of closed tunnel in Xiongqin (Xiongan to Qinzhou), as shown in Figure 10. The goal is still to image the wire 100 m away from the system prototype; the diameter of the wire is 0.5 cm. The difference is that the tunnel environment is relatively dim, and because it is still in the construction stage, there is still a lot of dust inside, such test conditions are designed to simulate the challenges that may be encountered in practical applications, focusing on the imaging ability of small targets at a distance. Thus, the imaging advantage of this system in a complex environment is demonstrated.

From the experimental environment, it can also be seen that the bad environmental conditions put forward higher requirements for imaging technology. Factors such as lighting and dust in real application scenarios have a significant negative impact on image quality compared to simulation scenarios. In such an environment, it is often difficult for conventional imaging techniques to capture clear and accurate images, resulting in a limited ability to identify and analyze distant targets, as shown in Figure 6b,d.

**Figure 11:**
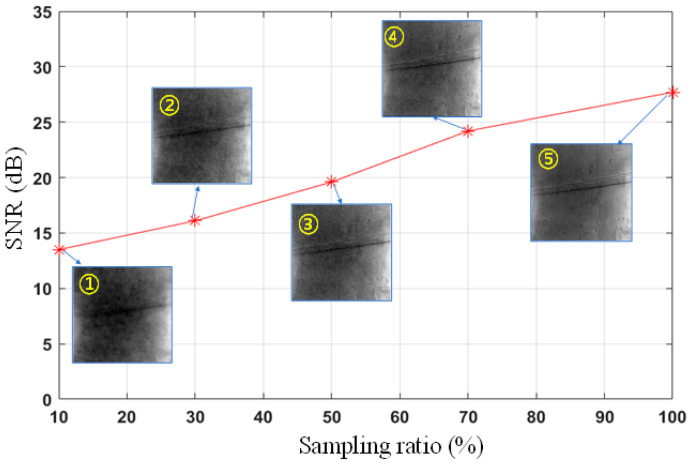
SNRs under different sampling ratios. The sampling ratios from ① to ⑤ were 10, 30, 50, 70, and 100%, respectively.

As emphasized in the experimental preparation, traditional imaging methods struggle to discern the cable targets. Under the same test conditions, our prototype successfully imaged distant wires, demonstrating the ability to maintain a high imaging quality in environments that are both dimly lit and have poor clarity, as shown in Figure 11. The corresponding imaging results at sampling ratios of 10%, 30%, 50%, 70%, and 100% are presented, with quantitative results showing SNR ratios of 13.5 dB, 16.1 dB, 19.6 dB, 24.2 dB, and 27.7 dB, respectively. This experiment revealed that under low-light and dusty conditions, our method is more conducive to capturing images of fine targets.

By comparing the imaging results under different sampling ratios, we found that under low sampling ratio conditions, single-pixel imaging technology combined with variable-resolution sampling and lateral inhibition characteristics could provide higher SNR ratios and clearer images. For instance, in an actual tunnel environment, the imaging result at a 10% sampling ratio had an SNR ratio of 13.5 dB, whereas the result at a 100% sampling ratio had a ratio of 27.7 dB. The experiments demonstrated that, even under the dim conditions inside the tunnel, the prototype system could successfully reconstruct recognizable images, which is crucial for guiding robots to avoid obstacles. This capability not only verified the robustness of the system but also showed its good environmental adaptability in maintaining relatively clear imaging under extreme conditions.

## 4. Conclusions and Future Works

The experimental results validated the effectiveness of our proposed eye-inspired variable-resolution single-pixel imaging technique for enhancing the environmental perception in special unmanned vehicles (UAVs). This innovative approach, tested extensively in both controlled indoor settings and real-world tunnel scenarios, demonstrated its potential for practical applications. In particular, in GPS-denied tunnel environments with dim lighting and dust, the technique showed a significant improvement in the efficiency and quality of image reconstruction for fine targets. The integration of lateral inhibition was also successful in reducing noise and artifacts, resulting in cleaner image reconstruction.

In future work, the primary focus will be on conducting more comprehensive field tests to confirm the robustness of the system under diverse environmental conditions. There is a clear need to develop advanced image-processing algorithms to achieve real-time imaging capabilities and to integrate this imaging system with autonomous robots or unmanned aerial vehicles (UAVs) for automated inspection tasks. Aligning technology with industry standards and regulatory requirements is also a priority for expediting commercial deployment. Our goal is to develop this technology into a reliable tool that can improve safety and efficiency in tunnel inspection and other applicable areas. By providing clearer and more accurate images than traditional imaging techniques, we hope to help inspectors spot potential problems faster so they can take preventive measures to avoid possible accidents and delays.” This will mark an important advance in imaging technology, providing more efficient and safer solutions for a variety of inspection and monitoring tasks.

## Data Availability

The data presented in this study are available upon request from the corresponding author.
